# Condensation heat transfer in microgravity conditions

**DOI:** 10.1038/s41526-023-00276-1

**Published:** 2023-04-04

**Authors:** Arianna Berto, Marco Azzolin, Stefano Bortolin, Marc Miscevic, Pascal Lavieille, Davide Del Col

**Affiliations:** 1 University of Padova, Department of Industrial Engineering, Via Venezia 1, 35131 Padova, Italy; 2 LAPLACE, Université de Toulouse, CNRS, INPT, UPS, Toulouse, France

**Keywords:** Mechanical engineering, Fluid dynamics

## Abstract

In the present paper, a thorough review of the experimental and numerical studies dealing with filmwise and dropwise condensation under microgravity is reported, covering mechanisms both inside tubes and on plain or enhanced surfaces. The gravity effect on the condensation heat transfer is examined considering the results of studies conducted both in terrestrial environment and in the absence of gravity. From the literature, it can be inferred that the influence of gravity on the condensation heat transfer inside tubes can be limited by increasing the mass flux of the operating fluid and, at equal mass flux, by decreasing the channel diameter. There are flow conditions at which gravity does exert a negligible effect during in-tube condensation: predictive tools for identifying such conditions and for the evaluation of the condensation heat transfer coefficient are also discussed. With regard to dropwise condensation, if liquid removal depends on gravity, this prevents its application in low gravity space systems. Alternatively, droplets can be removed by the high vapor velocity or by passive techniques based on the use of condensing surfaces with wettability gradients or micrometric/nanometric structuration: these represent an interesting solution for exploiting the benefits of dropwise condensation in terms of heat transfer enhancement and equipment compactness in microgravitational environments. The experimental investigation of the condensation heat transfer for long durations in steady-state zero-gravity conditions, such as inside the International Space Station, may compensate the substantial lack of repeatable experimental data and allow the development of reliable design tools for space applications.

## Introduction

In recent years, increasing interest has been addressed to the study of two-phase flow heat transfer in thermal control, thermal management and life support systems for space applications. Single-phase heat transfer systems may be progressively replaced by two-phase counterparts owing to the need of reducing the size and the weight of thermal management subsystems while meeting the increasing power requirements and heat dissipation demands of space aircrafts^1^. As the spacecraft is under the continuous influence of varying gravity conditions during its journey to space, the heat dissipation systems inside space vehicles are required to operate efficiently in different gravity environments.

During phase change processes such as condensation and flow boiling, the level and the direction of gravity acceleration strongly influence the spatial distribution of the liquid and vapor phases, having different densities^2^. As gravity is suppressed, a new balance between inertial, viscous and interfacial forces becomes effective and the mechanisms governing the interactions between phases drastically change^3,4^.

Considering filmwise condensation, only limited information is available for characterizing the fluid flow behavior and the condensation heat transfer in reduced gravity environments owing to the limited access to the existing platforms which allow to reproduce microgravity conditions and the inherent difficulties of performing experiments in such conditions. Among the available platforms for microgravity experiments (parabolic flights, drop towers, sounding rockets, International Space Station), parabolic flights represent the most widely used due to the affordable cost, the possibility to install large experimental racks and to interact manually with them, and the good data repeatability, despite the low quality of residual gravity (±0.01 *g*_*s*_). In adiabatic conditions, several experimental and theoretical studies have been performed to investigate the effect of the varying gravity level on the two-phase flow features^5–7^. Such studies were typically carried out in conventional tubes (inner diameter larger than 10 mm) during air-water co-current upward annular flows, using parallel wires conductance probes for the measurement of the liquid film thickness inside channels^8,9^. The available literature on this topic shows little coherence with regard to the effect of gravity on the film thickness and interfacial waves characteristics, which is found to be negligible for some authors^9^ and remarkable for others^8,10,11^. Such contradictory results suggest that further experimental studies are required during both adiabatic and diabatic two-phase flow, especially for the development of reliable predictive tools in reduced gravity environments or for updating existing correlations, which are typically retrieved from on-ground experiments^1,12–14^.

With regard to dropwise condensation, no experimental studies have been performed in microgravity conditions to the authors’ knowledge. Indeed, gravity is paramount in standard dropwise condensation for the removal of droplets which grow in size or coalesce with other droplets. Therefore, other experimental approaches for droplets removal need to be developed for exploiting the merits of dropwise condensation in low gravity space systems.

In the present work, a comprehensive survey of the available experimental and numerical studies dealing with in-tube condensation, condensation on enhanced surfaces and dropwise condensation in microgravity conditions is performed. Predicting methods for in-tube condensation heat transfer in microgravity conditions are also mentioned in the present paper. To conclude, the challenges and future perspectives of the research on condensation heat transfer under microgravity are outlined at the end of the paper.

### FILMWISE CONDENSATION INSIDE CHANNELS AND ON PLAIN SURFACES

Extensive experimental and theoretical research has been performed to investigate the influence of gravity on the interfacial behavior and flow condensation heat transfer by varying the tube inclination angle during on-ground experiments^15–19^ (see Table 1). When stratified smooth or stratified wavy flow occurs, the liquid is drained at the bottom of the tube due to gravity and a thin film of condensate is formed at the top of the tube, leading to an increase in the heat transfer coefficient^20^. When the flow regime changes to churn, intermittent or annular flows, the liquid phase sporadically or entirely covers the inner circumference of the channel, thus increasing the local thermal resistance and consequently decreasing the condensation heat transfer rate^21^. In such conditions, the condensation heat transfer coefficient can be enhanced by increasing the mass flux in order to get thinner liquid films. Whatever the channel orientation, in the absence of gravity the stratification of the two-phase flow does not occur. This has direct implications on the characteristics of the interfacial structures, on the liquid film thickness and, hence, on the condensation heat transfer.

**Table 1:** Summary of the works investigating the effect of gravity on filmwise condensation heat transfer in terrestrial applications.

Investigators	Type of study	Geometry	Fluids	Working conditions	Activities performed	Main results
Azzolin et al.^64^	Experimental and analytical	Circular 3.38 mm ID channel	R134a	Horizontal and vertical downflow *T*_sat_ = 40 °C Δ*T*_sat-wall_ = 3.1–17.1 K *G* = 50–200 kg m^−2^ s^−1^	HTC measurements Flow visualizations	The HTC in vertical downflow can be as low as half the value in horizontal flow at the same operating conditions.
Chang et al.^91^	Analytical	Semi-circular tube with 0.2 m diameter	Saturated steam	Horizontal Δ*T*_sat-wall_ = 5–25 K	Analytical study on laminar FWC over a horizontal semi-circular tube	If the capillary force is greater than the gravity force, the condensate is sucked into the two-phase zone leading to lower liquid film thickness and higher HTC.
Da Riva and Del Col^31^	Numerical	Circular 1 mm ID minichannel	R134a	Horizontal and vertical downflow *T*_sat_ = 40 °C *T*_wall_ = 30 °C *G* = 100, 800 kg m^−2^ s^−1^ *x* = 0.4–1	Numerical simulations of FWC inside a circular channel by means of VOF method	At *G* = 100 kg m^−2^ s^−1^, HTCs obtained in the horizontal configuration nearly doubles the one in vertical downflow due to stratification effects.
Del Col et al.^21^	Experimental and analytical	Square channel with 1.23 mm hydraulic diameter	R134a, R32	Horizontal, vertical and inclined (downflow and upflow) *T*_sat_ = 40 °C *G* = 100–390 kg m^−2^ s^−1^ *x* = 0.2–0.9	HTC measurements	Whatever the fluid, the effect of the channel inclination on the condensation HTC becomes noteworthy in downflow, at *x* < 0.6 and mass velocities lower than a critical value (*G*_crit_ = 150 kg m^−2^ s^−1^ for R134a, *G*_crit_ = 200 kg m^−2^ s^−1^ for R32). By applying the Buckingham theorem, a criterion is developed to predict the mass velocity at which the channel inclination starts to affect FWC.
Ewim et al.^18^	Experimental	Circular 8.38 mm ID channel	R134a	Horizontal, vertical and inclined (downflow and upflow) *T*_sat_ = 40 °C Δ*T*_sat-wall_ = 1–10 K *G* = 50–100 kg m^−2^ s^−1^ *x* = 0.1–0.9	HTC measurements Flow visualizations	The minimum HTC is found at the maximum temperature difference at an inclination angle of −90° (vertically downwards flow).
Lips and Meyer^15^	Review of numerical, analytical and experimental works	Circular channels (1.94–102.26 mm ID)	Steam, refrigerants	Horizontal, vertical and inclined (downflow and upflow)	Review of studies dealing with flow modeling of inclined two-phase flows in terms of flow pattern maps, void fractions, HTCs and pressure drops	The inclination angle influences the condensation HTC. Under certain conditions, an inclination angle may exist which leads to the optimum HTC.
O’Neill et al.^40^	Experimental and analytical	Circular 11.89 mm ID channel	FC-72	Horizontal, vertical downflow and upflow *P*_sat_ = 99.73–205 kPa *G* = 116.80–676.83 kg m^−2^ s^−1^	Development of mechanistic criteria based on dimensionless parameters and of a separated flow model to predict the mass velocity required for gravity independent flow during FWC	The minimum mass velocity required to overcome body force effects on FWC of FC-72 is around 420 kg m^−2^ s^−1^. The results from the developed predicting tools are in good agreement with the experimental results.
O’Neill et al.^22^	Experimental	Circular 7.12 mm ID channel	FC-72	Horizontal, vertical upflow and downflow *P*_sat_ = 130–160 kPa *G* = 50–350 kg m^−2^ s^−1^	Analysis of pressure oscillations during FWC Flow visualizations	Vertical upflow exhibits the most significant oscillatory behavior, although in its maximum case amplitude is only seen to be 7.9% of time-averaged module inlet pressure, indicating there is little safety risk posed by oscillations under considered operating conditions.
O’Neill et al.^19^	Experimental and analytical	Circular 7.12 mm ID channel	FC-72	Horizontal, vertical downflow and upflow *P*_sat_ = 127–132.1 kPa *G* = 50.3–360.3 kg m^−2^ s^−1^	HTC measurements Assessment of available predicting correlations	At low mass velocities vertical upflow exhibits the highest values of HTC while, as mass velocity is increased, results obtained for all three orientations begin to converge.
Olivier et al.^16^	Experimental and analytical	Circular 8.38 mm ID channel	R134a	Horizontal, vertical and inclined (downflow and upflow) *T*_sat_ = 40 °C *G* = 100–400 kg m^−2^ s^−1^ *x* = 0.1–0.9	HTC measurements Flow visualizations Void fraction measurements	Void fractions and HTCs increase with downward inclination angles with an optimum angle between −10° and −30° (downward flow). The void fraction and flow pattern map predictions are inadequate for inclined flow conditions.
Park et al.^17^	Experimental	Circular 11.89 mm ID channel	FC-72	Horizontal, vertical downflow and upflow *P*_sat_ = 102–171.93 kPa *G* = 13.32–343.79 kg m^−2^ s^−1^	HTC measurements Flow visualizations	At low G values, the three orientations yield significant differences in heat transfer performance; at high G values the local HTCs tend to converge for the three orientations.
Toninelli et al.^20^	Experimental and numerical	Circular 1 mm and 3.4 mm ID channels	R134a	Horizontal *T*_sat_ = 40 °C *T*_wall_ = 30 °C *G* = 100 kg m^−2^ s^−1^ *x* = 0.6	HTC measurements Flow visualizations Numerical simulations of FWC by means of VOF method	The gravity effect on the liquid distribution is significant for the 3.38 mm tube, but the HTCs are smaller compared to 1 mm minichannel due to the higher average liquid film thickness.

The main experimental and numerical results concerning fluid flow characterization and heat transfer during filmwise condensation (FWC) in the absence of gravity are listed in Table 2 and discussed in the following sub-sections. In Tables 1 and 2, *T*_sat_ is the saturation temperature, *P*_sat_ is the saturation pressure, *T*_wall_ is the wall temperature, *ΔT*_sat-wall_ is the saturation-to-wall temperature difference, *G* is the mass velocity, *ṁ*_*f*_ is the mass flow rate, Re_*G*_ is the Reynolds number of the gas phase, calculated using Eq. (1), while *x* is the mean/local vapor quality.

1 $${{{\mathrm{Re}}}}_G = \frac{{\rho _G \cdot u_G \cdot D}}{{\mu _G}}$$

**Table 2:** Summary of papers addressing filmwise condensation heat transfer in microgravity conditions.

Investigators	Type of study	Geometry	Fluids	Working conditions	Activities performed	Main results
Azzolin et al.^27^	Experimental (parabolic flight)	Circular 3.38 mm ID channel	HFE-7000	*T*_sat_ = 44.2–47 °C *G* = 70–170 kg m^−2^ s^−1^ *x* = 0.1–1	HTC measurements Flow visualizations	HTC decrease by 20% under microgravity compared to normal gravity at *G* = 70 kg m^−2^ s^−1^. Comparable HTCs during microgravity and normal gravity at *G* = 170 kg m^−2^ s^−1^. Annular flow observed under microgravity.
Barakhovskaia and Marchuk^66^	Numerical	Axisymmetric curvilinear fin with 9.62 mm height and 4 mm radius of the fin’s base	HFE-7100	Natural convection *T*_sat_ = 53 °C *T*_wall_ = 10–45 °C	Numerical study of FWC on a fin surface designed to guarantee a constant gradient of capillary pressure	With the selected fin shape the liquid film on the fin is thick enough for the experimental measurements with an optical system. The condensate film thickness along the fin under terrestrial gravity is half of the one obtained in absence of gravity.
Berto et al.^28^	Experimental (parabolic flight) and analytical	Circular 3.38 mm ID channel	HFE-7000	*T*_sat_ = 38–41.3 °C *G* = 30–50 kg m^−2^ s^−1^ *x* = 0.1–0.9	HTC measurements Liquid film thickness measurements Flow visualizations Statistical and spectral analysis of the interfacial waves Comparison with predicting models	Strong penalization of condensation heat transfer by 50–70% under microgravity. The frequency of the waves becomes higher with the increasing gravity level and mass flux. Mean absolute deviation between predicted HTCs/film thickness data and experimental values lower than 20%, but the dependence on vapor quality is not accurately foreseen.
Bortolin et al.^69^	Collection of experimental results from ESA ENCOM project (parabolic flight)	Axially symmetrical curvilinear fin with 15 mm height	HFE-7100	Natural convection *T*_sat_ = 51 °C	Liquid film thickness measurements Flow visualizations	Maximum local HTC detected at the tip of the fin and at the conjugation area of condensed film and the meniscus at the bottom of the fin.
Brendel et al.^23,24^	Experimental (parabolic flights)	Fin-and-tube condenser (6.2 mm ID, 16.8 m total coil length) in a vapor compression cycle	R134a	*T*_sat_ = 25.6–31.6 °C *G* = 15.2–35.1 kg m^−2^ s^−1^	Flow visualizations Check of the stability of the system	Condensation pressure rise by 3% under microgravity compared to normal gravity.
Chen et al.^4^	Review of numerical, analytical and experimental works	Flat surfaces, microgrooves, smooth and microfinned channels, round and rectangular minichannels	Steam and refrigerants		Assessment of the influence of gravity, shear stress, surface tension, and capillary and centrifugal forces on condensation heat transfer	Large potential for the use of vapor shear to remove the condensate in space applications. Surface tension and capillary forces can play a significant role in two-phase systems for space by properly tuning the shape and size of the channel.
Chow and Parish^92^, Faghri and Chow^30^	Numerical	Circular channel	Saturated steam	*P*_sat_ = 1 atm *T*_wall_ = 70, 85 °C Δ*T*_sat-wall_ = 15–30 K Re_*G*_ = 1000–50,000	Numerical study to investigate the effect of suction and vapor shear during in-tube condensation of vapor in a microgravity environment	Simultaneous suction and vapor shear can effectively drain the condensate to ensure the continuous operation of space condensers.
Da Riva and Del Col^31^	Numerical	Circular 1 mm ID minichannel	R134a	*T*_sat_ = 40 °C *T*_wall_ = 30 °C *G* = 100, 800 kg m^−2^ s^−1^ *x* = 0.4–1	Steady-state simulations of FWC using VOF method	The heat flow rate at *G* = 100 kg m^−2^ s^−1^ is 5% higher in vertical downflow as compared to zero-gravity. At *G* = 800 kg m^−2^ s^−1^ the HTCs in normal gravity horizontal, normal gravity vertical and zero-gravity simulations are comparable.
Glushchuk et al.^68^	Experimental (parabolic flight)	Curvilinear brass fin of 16 mm height	HFE-7100	Natural convection *T*_sat_ = 51 °C	HTC measurements Film thickness measurements Optical afocal system for flow visualizations	The local Nusselt numbers in hypergravity are about 10 times higher than those obtained under microgravity.
Glushchuk et al.^70^	Experimental (parabolic flight)	Cylindrical brass fin with 8.9 mm height and 5.9 mm diameter of the cylindrical part	HFE-7100	Natural convection *T*_sat_ = 45–55 °C	Heat flux measurements Film thickness measurements Optical afocal system for flow visualizations	Under reduced gravity, the highest heat fluxes are obtained at the corner of the fin tip, where the curvature gradient is maximum, and on its cylindrical part.
Gu et al.^35^	Numerical	Circular 1 - 4.57 mm ID minichannels	R1234ze(E)	*T*_sat_ = 40 °C *T*_wall_ = 30 °C *G* = 300–600 kg m^−2^ s^−1^ *x* = 0.4–0.9	Numerical study of FWC inside horizontal mini/micro-channels by means of VOF method	Gravity effect negligible for *D* = 1 mm even at *G* = 300 kg m^−2^ s^−1^, but of great importance for *D* = 2 mm and *D* = 4.57 mm.
Lee et al.^1,2^	Experimental (parabolic flights) and analytical	Circular 7.1 mm ID channel	FC-72	*T*_sat_ = 60–63.4 °C *G* = 38–472.3 kg m^−2^ s^−1^ *x* = 0.1–1	Local HTC measurements Flow visualizations A control-volume-based model of the condensation film is proposed	The influence of gravity is significant at low mass velocities, where the film in microgravity is smooth-laminar and circumferentially uniform in thickness. Kim and Mudawar^61^ model predicts the condensation HTCs in microgravity within 21%.
Li et al.^32^	Numerical	Circular 3.78 mm ID channel	R410A	*T*_sat_ = 46.85 °C *T*_wall_ = 36.85 °C *G* = 307, 720 kg m^−2^ s^−1^ *x* = 0.45–0.95	Numerical study of FWC inside a horizontal channel by means of VOF method	The mass transfer rate at zero gravity is much lower than that in normal gravity. Under zero-gravity the condensation process is mainly dominated by shear force and surface tension.
Li et al.^37^	Numerical	Square minichannel with 1 mm side length and different channel lengths (*L* = 50, 600 mm)	R134a	*T*_sat_ = 40 °C *G* = 50, 500 kg m^−2^ s^−1^ *x* = 0.3–1	Numerical study of FWC inside a horizontal square channel by means of VOF method	In short channels (50 mm long), the distribution of the liquid film is the same with and without gravity acceleration. In long channels (600 mm long) the flow is stratified under normal gravity and annular when gravity is ignored.
Marchuk et al.^38^	Numerical	Circular 1–5 mm ID channels with length from 100 to 200 mm	Pure ethanol (99.8%)	*P*_sat_ = 440 mbar *G* = 1.66 kg m^−2^ s^−1^	Numerical simulations of laminar FWC inside a circular tube by means of a finite-element method	Under microgravity the gravity force is weak and the surface tension forces decrease with the growth of the tube diameter.
Marchuk and Kabov^67^	Numerical	Disk-shaped fin (61.25 mm radius of the disk)	FC-72	*T*_sat_ = 56.7 °C Δ*T*_sat-wall_ = 5 K *ṁ*_*f*_ = 0.012 kg min^−1^	Numerical simulations of FWC on disk-shaped fin considering capillary forces	Due to the additional curvature of the disk-shaped fin, the condensate outflow is greater for the disk-shaped fin compared to a straight one.
Miscevic et al.^36^	Numerical	Circular channel with 560 μm inner diameter	N-pentane	*T*_sat_ ≈ 36 °C *G* = 3–15 kg m^−2^ s^−1^	Development of a 1-D model of convective condensation in the absence of gravity inside a capillary-driven two-phase loop	The predicted mean void fraction and condensation zone length are in good agreement with the experimental ones, taken during on-ground tests.
Mishkinis and Ochterbeck^63^	Analytical	3.34–16 mm ID channels	Ammonia, R134a, propylene	*T*_sat_ = 40 °C Δ*T*_sat-wall_ = 5–30 K *G* = 58–390 kg m^−2^ s^−1^	Detailed analysis of recent models and experimental data for condensing two-phase flow regimes, pressure gradients and local HTCs under Earth-normal gravity and microgravity	On-ground models developed for two-phase annular flow, such as Shah^58^ model, can be suitably extrapolated to the microgravity c*ase*.
Mudawar^41^	Review of experimental (parabolic flights) and numerical works	Channels with various cross-sectional shapes	Refrigerants		Review of published literature concerning reduced gravity flow boiling and flow condensation mechanisms, as well as predictive tools	Considering the scarcity of experimental studies on FWC under reduced gravity, long duration, steady-state heat transfer measurements on the ISS are needed to develop reliable design tools.
Nebuloni and Thome^39^	Numerical	Circular (1 mm diameter), square (1 mm side) and equilateral triangular (1 mm side) channels	R134a	*T*_sat_ = 24 °C *T*_wall_ = 23 °C	Numerical simulations of FWC inside different channel shapes to evaluate local/mean HTCs, liquid film thickness and void fraction	The HTC enhancement due to surface tension is clearly visible for square and triangular channels, while the gravity effect is non-negligible for the circular tube.
Reinarts et al.^25^	Experimental (parabolic flight) and analytical	Circular 8.7 mm ID channel	R12	*P*_sat_ = 5.45–5.65 bar	HTC measurements Flow visualizations	Decrease by 26% in the condensation heat transfer in the transition from normal gravity to microgravity.
Reinarts et al.^26^	Review of numerical, analytical and experimental works	Straight circular-cross section tubes	Single to multi-components refrigerants	Co-current two-phase flow	Review of works dealing with testing and modeling of flow regime transitions, pressure drops and condensation heat transfer under microgravity	Literature on FWC under microgravity mainly focused on annular flow regime, instead of slug/bubbly flow. Flow regime and pressure drop models for slug/bubbly flow are largely empirical and obtained considering air-water mixtures.
Sunden and Fu^3^	Review of numerical, analytical and experimental works	Flat surfaces, channels, cryogenic tanks			Review of capillary and two-phase flow, bubble dynamics, boiling and condensation heat transfer and solidification under microgravity	As the buoyancy forces become insignificant, other forces become important, such as capillary, viscous and electromagnetic forces. The simplest way to remove the condensate in a reduced gravity environment is vapor shear.
Toninelli et al.^34^	Numerical	Circular and square channels with 1 mm hydraulic diameter	R134a	*T*_sat_ = 40 °C *T*_wall_ = 30 °C *G* = 100 kg m^−2^ s^−1^ *x* = 0.4–1	Numerical simulations of in-tube FWC by means of VOF method	In the square channel the absence of gravity is not responsible for a heat transfer penalization compared to normal gravity as surface tension is the dominant force.
Zhang and Li^67^	Numerical	Circular 0.25–4 mm ID channels	R410A	*T*_sat_ = 46.85 °C *T*_wall_ = 36.85 °C *G* = 400, 1000 kg m^−2^ s^−1^ *x* = 0.25–0.95	Numerical simulations of FWC inside horizontal tubes by means of VOF method	The gravity effect on HTC is only noticeable at low vapor quality and mass flux in the larger tube diameter.

In Eq. (1) *ρ*_*G*_, *u*_*G*_ and *μ*_*G*_ are, respectively, density, velocity and dynamic viscosity of the gas phase, while *D* is the hydraulic channel diameter.

### Experimental studies

The precise knowledge of how changes in the gravity level affect the condenser operation is imperative for the accurate design of thermal management systems for space applications. Analysis of pressure signals in terrestrial condensing systems reveals the presence of oscillatory phenomena due to transient flow which can be encountered also in microgravity and hypergravity conditions and may impact the thermal performance of the cooling devices^22^. The stability and the performance of a vapor compression cycle working with R134a under varying gravity accelerations were experimentally investigated by Brendel et al.^23,24^ during parabolic flight campaigns. The onset of microgravity was found to cause a steep increase of the condensation pressure up to 3% due to the reduced liquid removal from the walls and the decreased heat transfer coefficients compared to normal gravity. Such instability issues and heat transfer penalization under microgravity were confirmed by Reinarts et al.^25,26^ during condensation tests with R12 inside a 8.7 mm inner diameter tube aboard the NASA KC-135 plane. In the transition from hypergravity to microgravity, the working fluid temperature and pressure at the inlet of the condensation test section were found to slightly increase and a significant decrease of the condensation heat transfer (in the order of 26%) was detected under microgravity conditions compared to normal gravity.

**Fig. 1:**
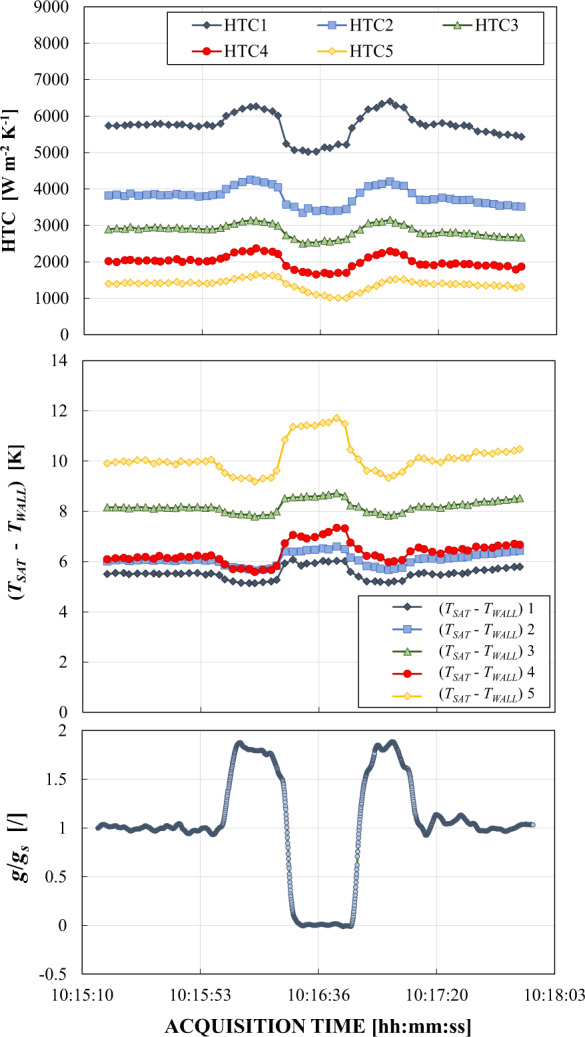
In-tube condensation heat transfer coefficient with the varying gravity level. Temporal variation of gravity acceleration, saturation-to-wall temperature difference and heat transfer coefficient of HFE-7000 during one parabola inside a 3.38 mm inner diameter channel at 130 kg m^−2^ s^−1^ (Azzolin et al.^27^).

The condensation heat transfer degradation in reduced gravity is well documented in the work by Azzolin et al.^27^: convective condensation of HFE-7000 inside a horizontal 3.38 mm inner diameter (ID) channel at mass flux *G* = 70–170 kg m^−^^2^ s^−1^ was studied during the 62^nd^ ESA Parabolic Flight Campaign. The employed test section was composed by two counter-current heat exchangers for quasi-local heat transfer coefficient measurement, separated by a borosilicate glass window for flow visualizations. Annular flow was observed under microgravity by means of a high speed camera, while stratified flows occurred during normal gravity and hypergravity conditions. Figure 1 reports the time recording of saturation-to-wall temperature difference and heat transfer coefficient in the five heat transfer sub-sectors for heat transfer measurements (numbering from 1 to 5 refers to decreasing vapor quality values) at *G* = 130 kg m^−2^ s^−1^. During microgravity, the saturation-to-wall temperature differences increase leading to smaller heat transfer coefficient values. The difference between the heat transfer coefficient measured in microgravity conditions and in normal gravity was found to be negligible at high mass flux (*G* = 170 kg m^−2^ s^−1^), while reaching 20% at small mass flux (*G* = 70 kg m^−2^ s^−1^). Similar conclusions were drawn in the works by Lee et al.^1,2^, where local heat transfer coefficients and flow pattern visualizations of FC-72 obtained inside a 7.1 mm inner diameter channel during a parabolic flight campaign are reported. Tests were performed under microgravity, Lunar (0.17 *g*_*s*_) and Martian gravity (0.377 *g*_*s*_) at mass flux from 129 to 341 kg m^−2^ s^−1^ and at 60–63.4 °C saturation temperature. The influence of gravity on flow pattern and condensation heat transfer was significant at low FC-72 mass fluxes. Lunar and Martian gravity caused the film to become thicker at the bottom of the tube and thinner at the top, leading to enhanced condensation heat transfer rates compared to microgravity conditions.

**Fig. 2:**
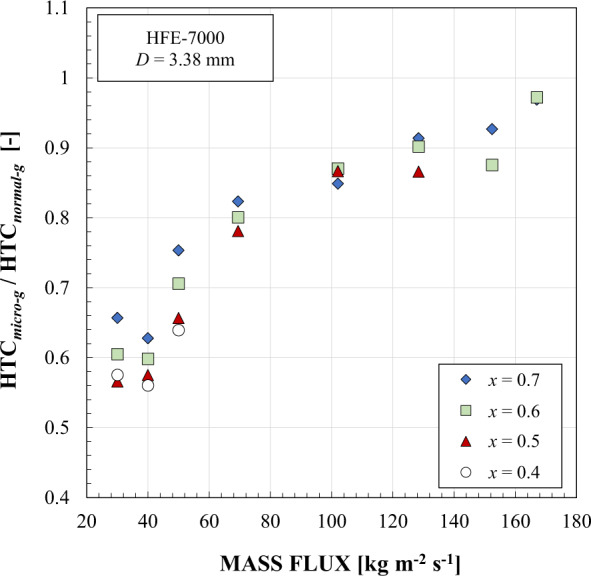
In-tube condensation heat transfer penalization under microgravity. Ratio of the experimental condensation heat transfer coefficient in microgravity to the one obtained in normal gravity conditions with HFE-7000 inside a 3.38 mm inner diameter channel at different mass fluxes *G* [kg m^−2^ s^−1^] and in the vapor quality *x* range from 0.4 to 0.7 (Berto et al.^28^).

With the aim to widen the experimental database of Azzolin et al.^27^, Berto et al.^28^ employed the same test section to perform condensation tests at low mass flux (from 30 to 50 kg m^−2^ s^−1^) during the 70th ESA Parabolic Flight Campaign. Simultaneous liquid film thickness and heat transfer measurements and flow pattern visualizations were performed during condensation using HFE-7000 as working fluid at 38–41 °C saturation temperature. The liquid film thickness was determined by coupling a shadowgraph technique (applied to the high speed camera images) to the local measurements performed by a chromatic confocal sensor and an interferometer. Similarly to the aforementioned experimental studies, gravity was found to enhance the condensation heat transfer especially with the decreasing mass flux. Indeed, as shown in Fig. 2, at *G* = 50 kg m^−2^ s^−1^ the heat transfer coefficient during normal gravity was about 33–56% higher than the one measured in microgravity conditions, while at *G* = 30 kg m^−2^ s^−1^ the heat transfer enhancement due to the gravity effect varied from 52% to 77% considering the whole range of vapor quality. Moreover, the heat transfer degradation in microgravity conditions was less noticeable at high vapor quality for all the tested mass velocities. A practical implication of these results is that it is possible to overcome the influence of gravity altogether by increasing the mass flux of the working fluid.

**Fig. 3:**
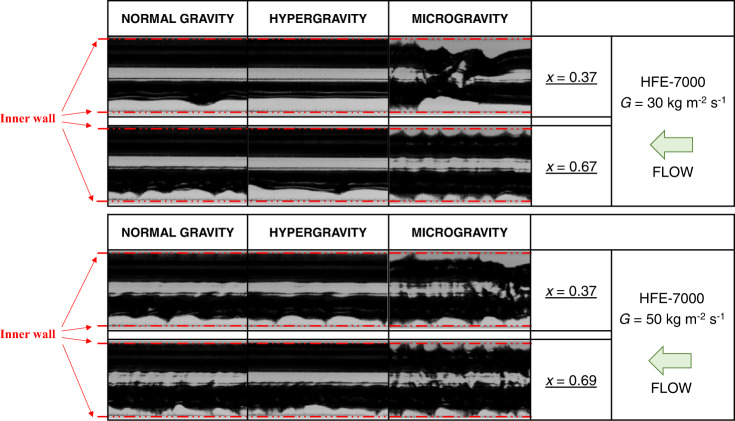
Flow pattern visualizations with the varying gravity level. Flow pattern visualizations during two-phase flow of HFE-7000 inside the 3.38 mm inner diameter channel at mass velocity *G* = 30 kg m^−2^ s^−1^ (top) and *G* = 50 kg m^−2^ s^−1^ (bottom) and different vapor qualities, performed in normal gravity, hypergravity and microgravity conditions (Berto et al.^28^).

In the work by Berto et al.^28^ the flow pattern visualizations, shown in Fig. 3, confirm stratified-wavy flow during normal gravity and hypergravity and annular-wavy flow in microgravity conditions. Moreover, during microgravity the interfacial features can be mainly classified as high-amplitude disturbance waves, which are sporadic (frequency around 10–20 Hz) and fairly regular in shape, and small-amplitude ripples, which are more frequent (frequency around 40–50 Hz). Instead, under normal gravity the interfacial waves display more random features in terms of frequency and velocity.

Considering the available experimental studies on filmwise condensation under reduced gravity, a potential improvement in the measuring accuracy could derive from the simultaneous local measurement of liquid film thickness and heat transfer coefficient, which was performed on-ground by Beaumale et al.^29^. In their work, two high-precision measurement techniques were applied to a 3.38 mm inner diameter sapphire tube allowing accurate measurement of the wall temperatures by means of an infrared camera and of the liquid film thickness using a confocal or an interferometric sensor. Moreover, some interesting results on condensation in reduced gravity could be obtained by testing various channel shapes (square, triangular, …), in order to address the balance between shear and capillary forces, and testing fluids that display different thermodynamic and transport properties.

All the aforementioned experimental studies agree that the condensation heat transfer penalization in reduced gravity can be overcome by increasing the vapor shear stress. Apart from vapor shear stress, the condensate can be efficiently removed in a microgravitational environment by means of suction through a porous wall, centrifugal force, electromagnetic field and capillary force^30^. Although these forces represent viable solutions for removing the condensate and increasing the condensation heat transfer, limited experimental and numerical studies have been conducted so far. A complete overview of the available studies is reported in Supplementary information (Section A).

Extensive research has been conducted on heat pipes in reduced gravity conditions, although such studies do not provide information specifically targeted to the condensation phenomenon. The available experimental and numerical works on this topic are briefly presented in the Supplementary information (Section B).

### Numerical and analytical studies

**Fig. 4:**
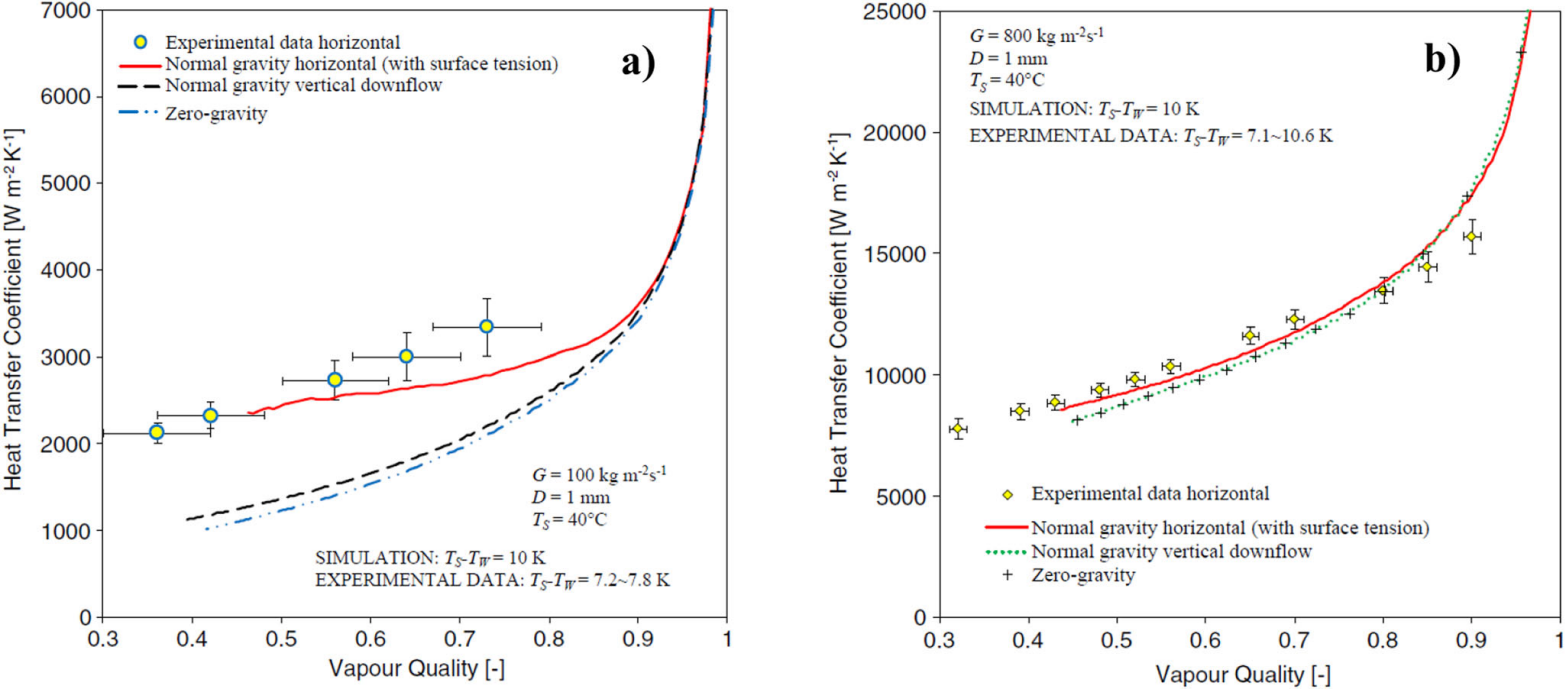
In-tube condensation heat transfer coefficients under zero-gravity resulting from numerical simulations, compared to horizontal and vertical configurations. Cross-sectional average heat transfer coefficients versus vapor quality at (**a**) *G* = 100 kg m^−2^ s^−1^ and (**b**) *G* = 800 kg m^−2^ s^−1^ during condensation inside a 1 mm inner diameter minichannel (Da Riva and Del Col^31^). The error bars correspond to the combined (Type A and Type B) experimental uncertainty.

The volume-of-fluid (VOF) method has been widely adopted in numerical studies to track the liquid-vapor interface during condensation. Transient simulations are typically performed to study interfacial waviness, intermittent flow patterns or transitions between flow pattern regimes, while steady simulations are mainly focused on steady liquid film thicknesses, vapor-liquid interface and local heat transfer coefficient. Da Riva and Del Col^31^ performed steady-state simulations of condensation of R134a inside a 1 mm ID circular minichannel in horizontal and vertical downflow configuration (with gravity) and in zero-gravity conditions by means of VOF method. Two different computational approaches were considered depending on the mass flux: the first approach for small mass fluxes (*G* = 100 kg m^−2^ s^−1^) corresponds to the assumption that the flow is laminar inside the liquid phase and turbulent in the vapor phase, whereas with the second approach for high mass fluxes (*G* = 800 kg m^−2^ s^−1^) the low-Reynolds SST *k* ~ *ω* model was adopted to account for turbulence in the liquid film. As depicted in Fig. 4, at *G* = 100 kg m^−2^ s^−1^ the condensation process is gravity dominated: the liquid condensed in the upper part of the tube is drained to the bottom by gravity, while the film thickness is kept uniform in the upper half of the tube. Due to stratification, 62% of the total heat transfer rate takes place at the upper half of the channel. Moreover, much higher heat transfer coefficients are obtained in the horizontal configuration as compared to the vertical one. The total heat flow rate at *G* = 100 kg m^−2^ s^−1^ is 5% higher in vertical downflow configuration as compared to the zero-gravity simulation: indeed, the gravity force acts together with the shear stress to slightly reduce the thickness of the condensate film. At *G* = 800 kg m^−2^ s^−1^, the effect of gravity is negligible since the condensate film is almost evenly distributed all around the internal circumference of the tube. In such condition, the normal-gravity horizontal, normal-gravity vertical and zero-gravity simulation cases display almost identical results.

**Fig. 5:**
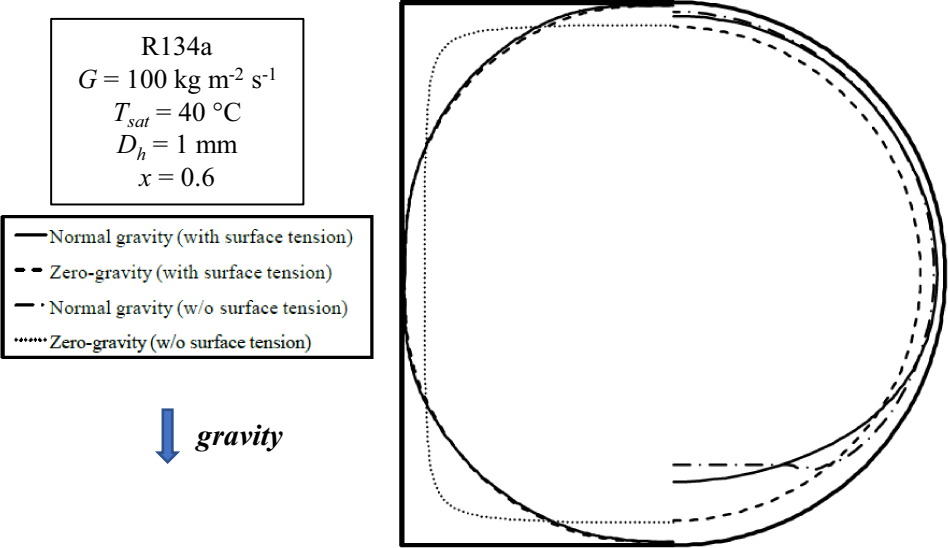
Effect of surface tension and gravity on the liquid phase distribution in square and circular channels. Liquid–vapor interface during condensation of R134a under capillary and gravity forces resulting from VOF simulations in circular and square minichannels at 40 °C saturation temperature, 100 kg m^−2^ s^−1^ mass flux and 0.6 vapor quality (Toninelli et al.^34^).

According to Li and co-workers^32,33^, gravity induces a heat transfer enhancement during condensation compared to zero-gravity condition, especially at low vapor quality and low mass flux inside large diameter tubes. This finding comes from numerical simulations performed using ANSYS Fluent with VOF method during condensation of R410A inside circular smooth tubes with inner diameter between 0.25 and 4 mm. Turbulent effects in the liquid and vapor flows during the condensation process were considered using the SST *k* ~ *ω* model, similarly to Da Riva and Del Col^31^. Similarly, Toninelli et al.^20,34^ concluded that the gravity effect on the condensation heat transfer and liquid phase distribution of R134a was more significant for a 3.38 mm inner diameter channel compared to a 1 mm minichannel. However, although the liquid film thinning at the top of the channel observed for the larger diameter tube due to gravity, the heat transfer coefficient in the 1 mm minichannel was higher than the one in the 3.38 mm tube due to the lower average liquid film thickness. Moreover, Toninelli et al.^34^ numerically found that the heat transfer coefficient inside a horizontal square 1 mm inner diameter channel in the absence of gravity is comparable to the one obtained under normal gravity conditions, since the capillary forces control the liquid film distribution in the channel and the influence of gravity is not significant. As shown in Fig. 5, in the square channel surface tension pulls the liquid towards the corners and a thin liquid film forms at the center of each flat side. Such distribution of the liquid film remains almost the same both under normal gravity and in the absence of gravity. Therefore, due to surface tension effects, the cross-sectional average condensation heat transfer coefficients inside the square minichannel are higher than those inside the circular one both with and without gravity effect.

The VOF numerical simulations of Gu et al.^35^ on R1234ze(E) condensation inside channels with inner diameter from 1 to 4.57 mm confirm the higher influence of gravity on heat transfer at low mass flux and low vapor quality inside large diameter tubes, already addressed by Li and co-workers^32,33^. At *G* = 400 kg m^−2^ s^−1^ and for a channel with hydraulic diameter *D*_*h*_ = 1 mm, the gravity effect was only noticeable at low vapor quality (*x* = 0.4), with a slightly thicker liquid film at the bottom of the tube compared to the top. When the diameter decreased to 2 mm, the liquid phase accumulated in the lower half of tube due to the increased gravity influence and decreased inertia effects, while generating a very thin film in the upper part of the tube wall. Instead, at *D*_*h*_ = 4.57 mm most of the liquid phase accumulated at the bottom of the tube. However, the authors observed that the average liquid film thickness is the same with and without gravity acceleration for each considered tube. Hence, gravity affects the condensation heat transfer by changing the liquid film distribution rather than the average film thickness. Since one of the viable ways to reduce the gravity effect on condensation heat transfer is to decrease the channel diameter, Miscevic et al.^36^ developed a 1-D model of convective condensation in the absence of gravity inside a capillary-driven two-phase loop of 560 μm inner diameter at low mass flux. The predicted mean void fraction and condensation zone length were found to be in good agreement with experimental data obtained during on-ground condensation tests, thus demonstrating the dominant role of capillary force upon flow.

Li et al.^37^ adopted the VOF method and the SST *k* ~ *ε* model for both the liquid and vapor phases to evaluate the effect of gravity on the condensation of R134a in a horizontal square minichannel with 1 mm hydraulic diameter and length *L* = 50, 600 mm at mass fluxes equal to 50 and 500 kg m^−2^ s^−1^. In the shorter channel, gravity exerts no significant effect on the condensation heat transfer, as the distribution of the liquid film inside the channel is the same with and without gravity acceleration. Instead, in the longer channel the liquid mainly resides at the bottom of the tube when gravity is present, while it is uniformly distributed inside the channel when gravity is ignored. Similar results were obtained by Marchuk et al.^38^ from a 3D film-vapor condensation theoretical model: the condensation heat transfer coefficient of pure saturated ethanol inside a 0.5 mm ID channel during microgravity was found to rapidly decrease with the increasing distance from the inlet of the tube due to the growth of the condensate film thickness around the whole perimeter of the tube.

The effect of gravity on the condensation heat transfer inside differently shaped channels was investigated by Nebuloni and Thome^39^. A theoretical model was developed to predict time dependent film condensation heat transfer for circular and non-circular (square and triangular) channel shapes under normal gravity and microgravity conditions. Surface tension effects led to the thinning of the liquid film towards the tube corners and to a heat transfer enhancement in the case of square and triangular shaped channels, while the effect of gravity was found to be relevant only for the circular channel.

It is worth highlighting that none of the aforementioned numerical and analytical studies involve a comparison with experimental data taken in reduced gravity platforms. This fact further stresses the need to enlarge the available experimental database on filmwise condensation for validating such numerical and analytical tools.

### Predicting methods for condensation heat transfer in microgravity conditions

Few predictive tools are available in the literature for identifying the flow conditions at which the gravity effect on the condensation heat transfer is negligible. Such methods are highly instrumental in the design of thermal management systems in aircraft and spacecraft avionics. Among them, Del Col et al.^21^ developed a dimensionless correlation for predicting the critical mass velocity at which the effect of inclination starts to appear during condensation inside tubes. The dimensionless inclination parameter Y* (Eq. 2), calculated as a function of the Eötvös number Eo (Eq. 3), the thermodynamic vapor quality *x*, the liquid and the vapor densities *ρ*_*L*_ and *ρ*_*G*_, allows to predict a gravity dependent region during condensation:

2 $${{{\mathrm{Y}}}}^ \ast = 0.185 \cdot {{{\mathrm{Eo}}}}^{0.35} \cdot \left( {\frac{{\rho _L - \rho _G}}{{\rho _G}}} \right) \cdot x^{ - 1.748}$$

3 $${{{\mathrm{Eo}}}} = \frac{{(\rho _L - \rho _G) \cdot g \cdot D_h^2}}{\sigma }$$

**Fig. 6:**
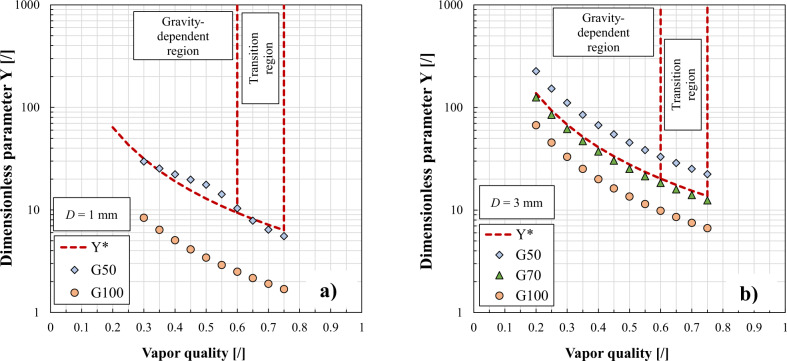
Range of mass fluxes showing an effect of inclination during in-tube condensation. Dimensionless inclination parameter reported versus vapor quality for HFE-7000 at 47 °C saturation temperature inside (**a**) a 1 mm inner diameter minichannel and (**b**) a 3 mm inner diameter tube (Azzolin et al.^27^).

In Eq. (3), *g* is the gravity acceleration (equal to 9.81 m s^−2^) and *σ* is the surface tension of the fluid. The Y* parameter should be compared with the dimensionless inclination parameter Y (expressed by Eq. 4), where *β* is the inclination angle which is equal to 0° in horizontal configuration and 90° in vertical downflow and (d*p/*d*z*)_*f,G*_ represents the single-phase pressure gradient for the vapor phase, calculated by Eq. (5):

4 $${{{\mathrm{Y}}}} = \frac{{(\rho _L - \rho _G) \cdot g \cdot \sin \left( \beta \right)}}{{\left( {\frac{{{{{\mathrm{d}}}}p}}{{{{{\mathrm{d}}}}z}}} \right)_{f,G}}}$$

5 $$\left( {\frac{{{{{\mathrm{d}}}}p}}{{{{{\mathrm{d}}}}z}}} \right)_{f,G} = \frac{{2 \cdot f \cdot (G \cdot x)^2}}{{D_h \cdot \rho _G}}$$

where *G* is the mass velocity and *f* is the friction factor. For a given operating condition, if Y is lower than Y*, no effect of inclination is predicted according to the criterion of Del Col et al.^21^. Azzolin et al.^27^ applied this criterion to round tubes of 1 mm and 3.38 mm inner diameter with HFE-7000 at 47 °C saturation temperature and the critical mass velocity was found to be respectively equal to 50 kg m^−2^ s^−1^ and 70 kg m^−2^ s^−1^, as shown in Fig. 6. This result confirms that, when the internal diameter becomes higher, the range of mass velocity displaying an effect of gravity is increased.

O’Neill et al.^40^ recently developed two mechanistic criteria based on dimensionless parameters for predicting the mass velocity required for gravity independent flow condensation heat transfer^41^. The first criterion addresses vertical downflow and vertical upflow, when gravity acts parallel to or opposite to the flow direction, respectively. The criterion is based on the Froude number Fr and vapor core Reynolds number Re_*c*_, which are respectively defined as follows:

6 $${{{\mathrm{Fr}}}} = \left[ {\frac{{\rho _G \cdot \left( {\bar u_G - u_i} \right)^2}}{{\rho _L \cdot g \cdot \sin \theta \cdot D_F}}} \right]$$

7 $${{{\mathrm{Re}}}}_c = \frac{{\rho _G \cdot \left( {\bar u_G - u_i} \right) \cdot \left( {D_h - 2\delta } \right)}}{{\mu _G}}$$

where *D*_*F*_ is calculated as follows:

8 $$D_F = \frac{{D_h^2 - \left( {D_h - 2\delta } \right)^2}}{{\left( {D_h - 2\delta } \right)}}$$

In Eqs. (6–8), *D*_*h*_ is the hydraulic diameter, *θ* is the orientation angle and *µ*_*G*_ is the dynamic viscosity of the liquid. Instead, $\bar u_G$, $u_i$ and $\delta$, which respectively represent the mean vapor core velocity, the interfacial velocity of the liquid film and the liquid film thickness, are all obtained using an annular flow control-volume-based model. This first criterion for gravity independence can be expressed with the following equation:

9 $$\left| {{{{\mathrm{Fr}}}}} \right| \,>\, \frac{{0.235}}{{a \cdot {\mathrm{Re}} _c^n}}$$

where *a* = 16 and *n* = −1 for 0 < Re_*c*_ < 2000, *a* = 0.079 and *n* = −0.25 for 2000 < Re_*c*_ < 20000 and *a* = 0.046 and *n* = −0.20 for Re_*c*_ > 20000.

The second criterion allows to determine the flow conditions at which horizontal flow condensation is able to guarantee circumferentially uniform annular flow. It is expressed in terms of Bond number Bo and Weber number We:

10 $$\frac{{\left| {{{{\mathrm{Bo}}}}} \right|}}{{{{{\mathrm{We}}}}^2}} \,<\, 5.12 \cdot 10^{ - 5}$$

where

11 $${{{\mathrm{Bo}}}} = \frac{{\left( {\rho _L - \rho _G} \right) \cdot g \cdot \cos \theta \cdot L_{char}^2}}{\sigma }$$

12 $${{{\mathrm{We}}}} = \frac{{\left( {\rho ^{\prime\prime} _L \cdot \rho ^{\prime\prime} _G } \right) \cdot \left( {\bar u_G - \bar u_L} \right)^2 \cdot L_{char}}}{{\left( {\rho^ {\prime\prime} _L + \rho^ {\prime\prime} _G } \right) \cdot \sigma }}$$

In Eqs. (11–12), $\bar u_L$ is the mean liquid velocity, $L_{char}$ is the characteristic length (equal to the hydraulic diameter *D*_*h*_ in the case of channels), while $\rho _L^{\prime\prime}$ and $\rho _G^{\prime\prime}$ are modified expressions of the liquid and vapor density, respectively^40^.

**Fig. 7:**
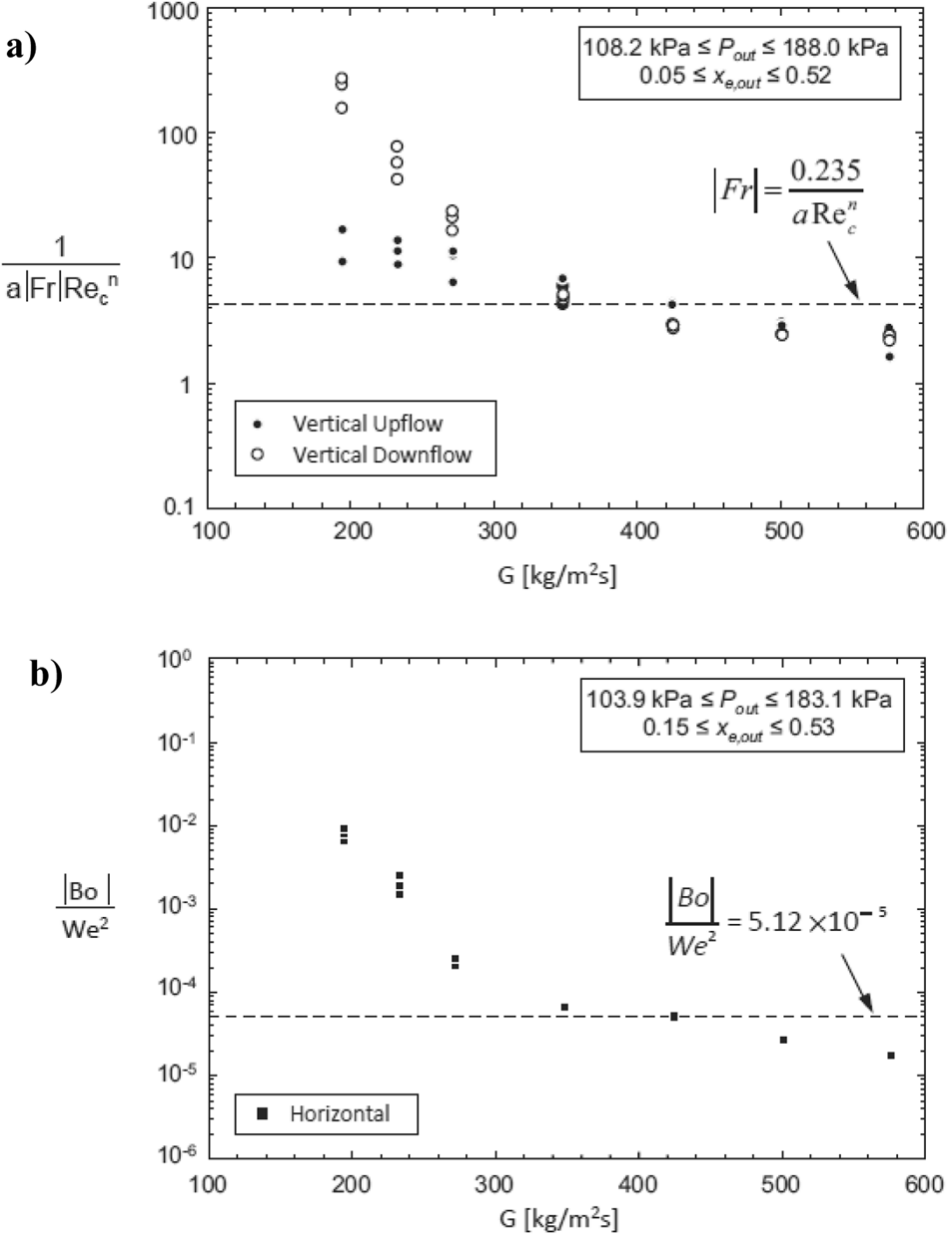
Mechanistic criteria for gravity-independent condensation heat transfer. Values of the dimensionless group in (**a**) the first criterion and (**b**) the second criterion for gravity-independent condensation heat transfer versus inlet mass velocity, evaluated using the experimental exit conditions (O’Neill et al.^40^).

The dimensionless criteria expressed by Eqs. (9) and (10) allow to determine the minimum mass velocity required to overcome body force effects on flow condensation heat transfer. Such criteria were developed considering the experimental heat transfer data obtained by Park et al.^17^ during condensation of FC-72 inside a 11.89 mm inner diameter channel in horizontal, vertical downflow and vertical upflow orientations. The authors observed that the local heat transfer coefficients for the three orientations overlap at mass velocities in the range *G* = 425–577 kg m^−2^ s^−1^. In Fig. 7, the values of the dimensionless group in the first and the second criterion are reported versus their respective mass velocity. Points below the dashed line indicate that the heat transfer performance of this configuration would be independent of gravity. The critical mass velocity of FC-72 is around 420 kg m^−2^ s^−1^, which is in good agreement with the experimental data.

Several predicting models for condensation heat transfer inside conventional and mini-/micro-channels in normal gravity conditions have been developed over the years. For conventional horizontal channels (*D*_*h*_ > 3 mm), condensation models differentiate between gravity-dominated flow regime, where the heat transfer coefficient is sensitive to the saturation-to-wall temperature difference driving force, and shear-dominated flow regime, owing to the influence of mass flux and vapor quality^42–47^. For mini-/micro-channels, since the gravity effect condensation is expected to be less important, correlations developed for condensation inside circular conventional tubes may not be able to accurately predict the heat transfer coefficient, especially at small mass flux^48–51^.

In a reduced gravity environment, the applicability of the available predicting tools for condensation heat transfer inside tubes, which are derived from experiments performed on ground, is not guaranteed and must be verified by means of experimental investigations. In the work of Berto et al.^28^, the models of Shah^47^, Cavallini et al.^52^ and Kosky and Staub^53^ were considered for the prediction of the condensation heat transfer coefficient of HFE-7000 during microgravity inside a 3.38 mm inner diameter tube at mass velocity from 30 to 50 kg m^−2^ s^−1^. Even if the mean absolute deviations were found to be smaller than 20%, none of the selected models was able to predict the data trend with vapor quality, whatever the mass velocity. The same conclusion was drawn considering the comparison between the experimental average liquid film thickness data and the values predicted by the models of Cioncolini et al.^54^, Cavallini et al.^52^ and Kosky and Staub^53^. Therefore, for such low values of mass flux, gravity plays a non-negligible role.

Kim and Mudawar^55^ developed a control-volume model for annular flow condensation. This model was adopted by Lee et al.^2^ for the prediction of the condensation heat transfer coefficient of FC-72 at mass velocity *G* = 129–340.5 kg m^−2^ s^−1^ inside a 7.1 mm inner diameter channel during microgravity. The model showed an acceptable agreement with the heat transfer coefficient data, with an overall mean absolute error MAE of 27.5% (60.5% and 97.4% of the data falling within ±30% and ±50%). Lee et al.^1^ compared the experimental data taken during condensation of FC-72 in microgravity in the 7.1 mm I.D. tube with the predictions of well-known correlations for the condensation heat transfer coefficient (Akers et al.^56^, Cavallini and Zecchin^57^, Shah^58^, Dobson and Chato^42^, Wang et al.^59^, Koyama et al.^60^, Kim and Mudawar^61^). The smallest mean absolute deviation between predicted and experimental heat transfer coefficients was obtained with Kim and Mudawar^61^ model (MAE = 21.8%, with 69.2% of points falling within ±30%), followed by the correlations of Shah^58^ (MAE = 27.6%) and Wang et al.^59^ (MAE = 28.9%). The success of the Kim and Mudawar^61^ correlation, indicated by Eq. (13), was attributed to its reliance on a massive database, which includes 17 different working fluids and a broad range of operating conditions and geometrical parameters.

13 $$\frac{{{{{\mathrm{HTC}}}} \cdot D_h}}{{\lambda _L}} = 0.048 \cdot {\mathrm{Re}} _L^{0.69} \cdot {\mathrm{Pr}} _L^{0.34} \cdot \frac{{\phi _G}}{{X_{tt}}}$$

In Eq. (13), *λ*_*L*_ is the liquid thermal conductivity, Re_*L*_ is the Reynolds number of the liquid phase (=*G* · (1 − *x*) · *D*_*h*_/*μ*_*L*_), Pr_*L*_ is the Prandtl number of the liquid (=*μ*_*L*_· *c*_*p,L*_/*λ*_*L*_) and *ϕ*_*G*_ is the two-phase multiplier, defined as follows:

14 $$\phi _G^2 = 1 + C \cdot X + X^2$$

while *X* is the Lockhart–Martinelli parameter, expressed as function of the frictional pressure gradients based on the actual flow rates for the individual phases:

15 $$X = \sqrt {\frac{{\left( {{{{\mathrm{d}}}}p/{{{\mathrm{d}}}}z} \right)_L}}{{\left( {{{{\mathrm{d}}}}p/{{{\mathrm{d}}}}z} \right)_G}}}$$

Reinarts et al.^25^ and Best et al.^62^ applied a modified Dittus-Boelter correlation, given by Eq. (16), for the prediction of the condensation heat transfer coefficient of R-12 in the low vapor quality region inside a 8.7 mm inner diameter channel during microgravity:

16 $$\frac{{{{{\mathrm{HTC}}}} \cdot D_h}}{{\lambda _L}} = 0.023 \cdot {\mathrm{Re}} _L^{0.8} \cdot {\mathrm{Pr}} _L^{0.3} \cdot \left( {1 + x \cdot \frac{{\rho _L}}{{\rho _G}}} \right)^{0.8}$$

The mean absolute deviation between experimental and predicted values was found to be lower than 31%. Mishkinis and Ochterbeck^63^ assessed the reliability of recent models for condensing two-phase flow regimes, pressure gradients and local heat transfer coefficients in normal gravity and microgravity conditions with ammonia, propylene and R134a. They concluded that on-ground models developed for two-phase annular flow, such as Shah^58^ model, can be suitably extrapolated to the microgravity case as the physical mechanisms are the same for terrestrial and microgravity annular flow conditions.

Further experimental investigations in microgravity are needed to assess existing 1-g correlations in microgravity applications or to develop new models for the prediction of the condensation heat transfer coefficient inside channels. Although annular flow is the main flow pattern observed during microgravity, the validity of available terrestrial correlations for two-phase annular flow heat transfer when applied for design purposes in a microgravity environment should be experimentally verified, especially at low mass fluxes due to increased effect of surface tension and viscous forces^64^.

## Filmwise condensation on enhanced surfaces

A widely employed method for enhancing filmwise condensation involves the creation of fins on the heat transfer surface. The heat transfer enhancement is provided not only by increasing the contact area between the vapor phase and the cold surface but also by the effect of surface tension forces, which redistribute the condensate along the fin surface together with other forces (gravity, shear stress, …), and by the induced turbulence in the liquid film during flow condensation inside channels.

If a liquid–vapor interface is curved, a gradient of capillary pressure is established across the film according to Laplace equation:

17 $$P_L - P_V = \frac{\sigma }{R}$$

where *P*_*L*_ is the pressure in the liquid film, *P*_*V*_ is the vapor pressure and *R* is the curvature radius of the film surface. Any curvature variation along the interface induces a pressure inhomogeneity within the liquid which leads to fluid redistribution. The fin shape also influences the distribution of the condensate film along the fin surface, hence it can be identified as one of the parameters that must be optimized during the design phase^65^. Barakhovskaia and Marchuk^66^ identified the most appropriate fin shape for condensation experiments under microgravity, able to guarantee a stable flow of the condensate film under the effect of a constant capillary pressure gradient. The condensate film thickness of HFE-7100 under terrestrial gravity was found to be half of the one obtained in the absence of gravity, leading to a much higher condensation rate. The designed fin shape was proposed also for experiments under microgravity onboard the ISS. Marchuk and Kabov^67^ developed a mathematical model for investigating filmwise condensation of FC-72 on a curvilinear disk-shaped fin under microgravity conditions, taking into account the influence of capillary forces.

Few experiments have been performed so far to investigate filmwise condensation on fins under microgravity. Glushchuk et al.^68^ conducted a condensation experiment of pure HFE-7100 on a curvilinear brass fin of 16 mm height aboard the Airbus A300 Zero-G aircraft for ESA Parabolic Flight campaigns. An optical afocal system was developed for measuring the temporal evolution of the condensate liquid film along the fin, which was in turn used to deduce the local Nusselt numbers in various gravity conditions. The derived heat of condensation was found to increase in hypergravity and decrease noticeably during microgravity. The thickness of the condensate film on a curvilinear surface of 15 mm height was measured with the same optical setup employed by Glushchuk et al.^68^ during a parabolic flight campaign^69^. Under microgravity, a local minimum in the liquid film thickness was detected at the conjugation area of condensed film and the meniscus at the bottom of the fin. Such liquid film distribution generates the two local maxima in the heat transfer coefficient values: the first one is located around the tip of the fin, while the second one is related to the conjugation area (where the film thickness is at its minimum).

**Fig. 8:**
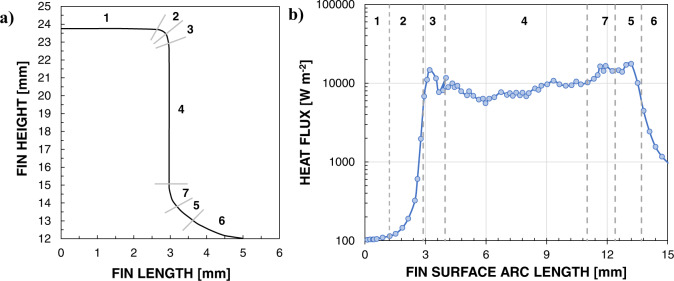
Condensation heat transfer on a fin surface under normal gravity. Heat flux distribution (**b**) under normal gravity for each selected area of the fin (**a**) (Glushchuk et al.^70^).

**Fig. 9:**
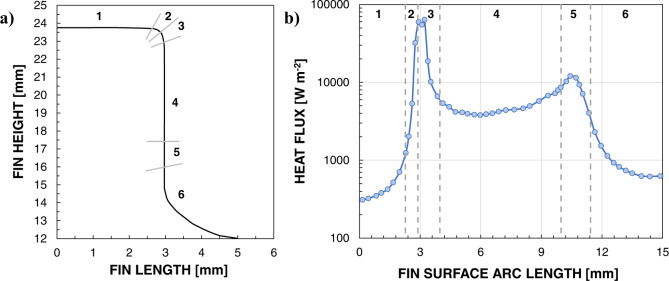
Condensation heat transfer on a fin surface under microgravity. Heat flux distribution (**b**) under microgravity for each selected area of the fin (**a**) (Glushchuk et al.^70^).

Glushchuk et al.^70^ experimentally analyzed the balance of forces acting on the condensate flow during condensation on a single cylindrical fin under various gravity conditions (0.05 *g*_*s*_, 1 *g*_*s*_ and 1.8*g*_*s*_). A round cylinder of 8.9 mm height with smoothed corners was chosen as the most suitable axisymmetric surface for measuring the liquid film thickness using an optical afocal system. During both normal gravity and microgravity phases, the liquid mainly accumulates at the fin tip and at the fin base leading to thicker condensate film under reduced gravity. Surface tension forces overcome gravity at both parts of the fin corner, in the area where the thin film at the lateral wall joins the liquid meniscus and when the liquid level at the fin base is very small. Analysis of the heat flux distribution along the fin surface under both normal gravity and microgravity (Figs. 8, 9) underlines that the areas under the dominant effect of surface tension forces display high values of heat flux, providing 10–15% of the total heat load. Under reduced gravity, the highest heat fluxes (in the order of 10–100 kW m^−2^) are obtained at the corner of the fin tip, where the curvature gradient is maximum, and on its cylindrical part.

## Effect of gravity during dropwise condensation

During dropwise condensation (DWC) the vapor phase changes to liquid phase forming discrete droplets on the surface whose temperature is below the dew temperature of the condensing fluid. It is well established that DWC allows to reach very high values of the heat transfer coefficient compared to filmwise condensation (FWC)^71^. The creation of surfaces that can promote DWC is one of the main issues. The available processes to treat the solid substrate and the fundamental mechanisms involved during DWC are described, for instance, in a recent paper by Wang et al.^72^. DWC is a cyclic process: condensation begins at a molecular scale with drops formation in preferred nucleation sites^73^. Growing by direct condensation at first and later by coalescence, drops reach the critical size at which external forces (e.g., gravity, vapor drag) overcome adhesion forces and they start to move, sweeping the surface and making new nucleation sites available.

To achieve DWC, the surface energy of the wall must be lower compared to the surface tension of the condensing fluid: for this reason, it is easier to promote DWC with high surface tension fluids such as water. Furthermore, since droplets after growing must be removed from the surface (this allows to clean the surface and re-start the nucleation process), high droplet mobility is required. As demonstrated by Cha et al.^74^, achieving stable DWC is not governed by surface intrinsic wettability (which is related to the static contact angle) but rather, it is dictated by low contact angle hysteresis. In fact, as shown in Parin et al.^75^, DWC can be achieved even on hydrophilic surfaces with advancing contact angle below 90°.

Since the cyclic DWC process is based on the removal of formed droplets, thus cleaning the surface and allowing the formation of new droplets, DWC experiments on ground are usually run on vertical surfaces in quiescent vapor conditions: when the gravity force overcomes the adhesion force, the droplet starts to move sweeping the surface.

The dependence on gravity for liquid removal may limit the potential application of DWC in low gravity environments and even on ground when considering horizontal surfaces. Gravity can affect DWC in different ways, depending on surface characteristics and vapor conditions. In the following, the effect of gravity during DWC on smooth surfaces and nanostructured surfaces is addressed. Available studies concerning the gravity effect during DWC are summarized in Table 3. There are no studies of DWC performed in microgravity.

**Table 3:** List of available studies that address the gravity effect during DWC.

Investigators	Type of study	Platform	Surface	Fluid	Activities performed	Main results
Bonner et al.^81^	Experimental	Two-phase thermosyphon, inclinable test section	Copper with self-assembled monolayer	Water	HTC measurements	Heat transfer coefficient on a horizontal surface of 35,000 W m^−2^ K^−1^ with a gradient surface.
Mancio Reis et al.^83^	Experimental	Condensing chamber with variable inclination of the sample	Silicon substrate with silanization	Water	HTC measurements Images of droplet population	Gradient wettability surface allows maintaining DWC regime, even where no gravity can be used as a mechanism for droplets removal. HTC found to be 3.4 times greater than the one on an untreated surface.
Mukherjee et al.^88^	Experimental	Humidity chamber, variable inclination of the sample	Nanopillared superhydrophobic surfaces (silicon substrate)	Humid air	HTC measurements Images of droplet population	Jumping droplets observed for different surface orientations. Gravity-assisted vertical orientation can increase the HTC by 100% compared to a horizontal orientation.
Talesh Bahrami and Saffari^77^	Analytical model	-	Micro/nanostructured inclined tube	Water	Numerical study of DWC on a micro/nanostructured inclined tube	Vertical tubes have five times higher overall heat transfer rate compared with the horizontal one.
Tancon et al.^79^	Experimental	Two-phase thermosyphon, vertical test section, downward vapor flow	Sol–gel coated aluminum sample and reduced graphene oxide coated copper sample	Water	HTC measurements Images of droplet population	For a sol–gel coated aluminum sample, increasing the vapor velocity from 3 m s^−1^ to 15.5 m s^−1^ leads to an increase of the HTC by about 40%.
Tancon et al.^80^	Analytical model	-	Flat surfaces with advancing contact angle < 90°	Water	HTC measurements Images of droplet population Modeling of DWC	Droplet departing radius decreased due to the drag force of the vapor. An equation is proposed to account for the steam velocity on the maximum droplet radius when vapor drag and gravity forces act in the same direction.
Wang et al.^76^	Experiments and lattice Boltzmann numerical simulations	Condensing chamber with adjustable inclination	Nanostructured copper superhydrophobic surface	Water	Flow visualizations HTC measurements Lattice Boltzmann simulations for coalescence induced droplet jumping	Surface inclination plays a determinant effect on the critical removal diameter of droplet. Compared to the horizontal surface, condensation heat transfer is enhanced by 72.4% in vertical configuration and Δ*T* = 2 K.
Xu and Chen^84^	Molecular dynamics simulations	-	Composite V-shaped surface with multi wettability gradients	Water	Numerical simulations by molecular dynamics	Condensation mode can be controlled by a composite V-shaped surface with multi wettability gradients, accelerating the condensate drainage both with and without gravity.

As stated before, the gravity force is often used to remove the biggest droplets, contributing to reduce the critical radius beyond which the drops are not pinned on the surface anymore. After departure, sliding droplets clean the surface promoting the nucleation of new small droplets. The reduction of the departing radius provides an increase of the condensation heat transfer coefficient since big droplets present the highest heat transfer resistance due to thermal conduction through the liquid. Wang et al.^76^ combined visual experiments and lattice Boltzmann numerical simulations to analyze, among others, the influence of inclination on the critical sliding radius and global heat transfer coefficient. The critical sliding radius is found to be 50% higher at 30° inclination with respect to 90° case (i.e., vertical orientation). Compared to a horizontal surface, the maximum heat transfer enhancement corresponds to 72.4% and it is obtained at 90° inclination. Talesh Bahrami and Saffari^77^ presented a numerical study of DWC on a micro/nanostructured surface finding that the orientation of the surface in the gravity field plays an important role on the drop size distribution. Since drops can oscillate during DWC (affecting the mobility or even causing the detachment of drops from the surface), Sakakeeny and Ling^78^ established, by means of numerical simulations, that the effect of gravity intensity on oscillation frequency is particularly significant when the contact angle is large (i.e., hydrophobic or superhydrophobic surface).

**Fig. 10:**
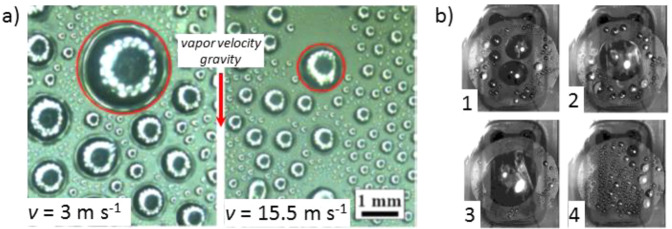
Effect of vapor velocity and wettability gradient surfaces on dropwise condensation. **a** Effect of the vapor velocity *v* on droplet departing radius during DWC of steam over a vertical surface, gravity level *g* = 9.81 m s^−2^ (Tancon et al.^79^). **b** Images of DWC over a horizontal surface with wettability gradient (Mancio Reis et al.^83^): droplets nucleate (1), grow (2), coalesce up to a certain radius (2–3); the biggest droplets are removed (3–4) towards the periphery of the treated zone and new droplets appear (4).

In the absence of gravity force and in presence of vapor flowing over the surface, the drag force of the vapor that acts on the droplets can be used to remove the droplets and thus to clean the surface, allowing stable DWC (Fig. 10a). Tancon et al.^79^ experimentally studied the effect of steam velocity during DWC on two different specimens vertically oriented: a sol–gel coated aluminum sample and a reduced graphene oxide coated copper sample. When varying the inlet vapor velocity in the range between 3 and 15.5 m s^−1^, due to the increase of the vapor drag force on the droplets, a reduction of the droplet departing radius was observed along with an increase of the condensation heat transfer coefficient. The same research group also proposed an analytical model for the estimation of the droplet departing radius in presence of non-negligible vapor velocity^80^.

In the absence of vapor flow, a passive mechanism can be used to remove droplets: Bonner et al.^81^ used a surface energy gradient (wettability gradient) on the condensing surface. The wettability gradient creates a difference in the contact angles along the perimeter of the condensing droplets, causing the motion of the droplets towards the surface regions with increased wettability. The movement of the droplets prevents flooding and allows the condensation of new droplets on the surface. Even when the solid substrate was placed vertically, the wettability gradient surface led to 35% higher heat transfer coefficient compared to a traditional DWC surface. Horizontally, the wettability gradient surface was able to maintain the dropwise regime. Gu et al.^82^ explained the heat transfer enhancement due to the wettability gradient with the fast motion of the droplets on the surface. Indeed, the motion of the droplets on a wettability gradient surface was found to be much less sensitive to gravity as the driving force is inherent in the energy of the surface. Mancio Reis et al.^83^ experimentally showed that the heat transfer coefficient during the transient regime (i.e., during about half an hour from the beginning of the condensation process) on a horizontal surface with a radial wettability gradient (Fig. 10b) is enhanced by a factor 2 to 3.4 (depending on the ageing of the surface) in comparison to an untreated surface (silicon in the raw state). Xu and Chen^84^ numerically studied the effect of gravity on nanometric-droplets condensation on a composite V-shaped surface with wettability gradients by means of the molecular dynamics approach. They concluded that the condensation rate becomes larger with the increasing gravity level.

When the surface includes micrometric or nanometric structuration, the droplets can wet (Wenzel state), partially wet (Wenzel-Cassie state) or not wet (Cassie state) the surface. In the Wenzel state, the droplet penetrates through micro/nanostructures and fully wets their walls and cavities; in the Cassie state, the droplet suspends over micro/nanostructures; in the partially wet state droplets locally wet the substrate between the pillars (liquid-filled nanostructures under a portion of the nominally Cassie droplet). Miljkovic et al.^85^ showed that partially wetting droplets led to a heat transfer rate 4-6 times higher than droplets in the Cassie state. Instead, the possible effect of gravity force on the droplet state is not well addressed in the literature. Xu et al.^86^ performed molecular dynamics simulations of the water wetting on an array of micro pillars under different gravity conditions. They found that a microgravity environment favored the transition from Wenzel state to Wenzel-Cassie or Cassie state.

When DWC involves superhydrophobic structured surfaces, the coalescence between two droplets can generate the jumping of the daughter drop^87^. When this occurs in microgravity, the jumping droplets may not go back to the solid surface. Mukherjee et al.^88^ used superhydrophobic nanopillars to passively decrease the maximum droplet departure radius and they studied the effect of orientation on the global heat transfer rate. Compared to the horizontal case, an enhancement of 40% is obtained for an inclination of 45°, and up to 100% for an inclination of 90°. In the case of jumping droplets, the heat transfer through each individual drop is limited because of the reduced contact area between the surface and the droplet. This can be partially overcome by considering biphilic surfaces, as suggested by Hoque et al.^89^.

It can be concluded that there is a lack of experimental studies concerning DWC in microgravity environments (parabolic flight, space station). The challenge, in addition to the production of more durable coatings, is to find efficient mechanisms (vapor drag force, wettability gradient surfaces, jumping droplet) and design tools to allow droplet removal in the absence of gravity and thus stable DWC.

## Challenges and future perspectives

Considering the most widely used platforms for reproducing microgravity (i.e., drop towers, sounding rockets, parabolic flights), condensation heat transfer experiments may fail to yield reliable and steady-state experimental data due to the limited duration of the microgravity phase.

Most of the available technical knowledge regarding the effect of gravity during condensation comes from experiments conducted in terrestrial environment by varying orientation. Such experiments are not sufficient to precisely simulate reduced gravity conditions owing to the inability to completely eliminate gravity components parallel or opposite to the flow direction.

Parabolic flight campaigns can enable the development of criteria for identifying the working conditions needed to achieve gravity-independent performance, but reliable design tools for condensation in microgravity require steady-state heat transfer data and flow visualizations for a longer duration. These experimental conditions can be achieved only with space experiments.

Considering filmwise condensation, further experiments are required for assessing the effect of gravity on the condensation heat transfer, especially at low mass fluxes, when the heat transfer coefficients are found to be strongly penalized by the absence of gravity. Moreover, condensation heat transfer coefficients and liquid film thickness data should be evaluated under various saturation-to-wall temperature differences, whose effect on the condenser performance has never been assessed before in a reduced gravity environment. The channel shape represents another key factor to counterbalance the detrimental effect of the absence of gravity on condensation heat transfer. Surface tension forces exert a great influence on the liquid film distribution especially for square or rectangular channels, whose behavior under reduced gravity has been addressed only in numerical studies. New experimental data on these topics would be essential for the validation of the existing numerical and analytical studies on condensation heat transfer in zero gravity conditions. With regard to dropwise condensation, experimental investigations under microgravity or zero-gravity conditions could help to validate the numerical simulation results and assess the reliability of the proposed practical solutions (i.e. wettability gradient surfaces) for aerospace applications.

At the present time, the Flow Boiling and Condensation Experiment (FBCE), resulting from the joint collaboration between the Purdue University Boiling and Two-Phase Flow Laboratory (BTPFL) and the NASA Glenn Research Center, is onboard the ISS^90^. The experiment aims at evaluating the influence of body force on two-phase transport phenomena in pursuit of mechanistic models as well as correlations, and determining the minimum flow criteria to ensure gravity-independent flow boiling and condensation. The European Space Agency (ESA) is currently involved through the SciSpacE (Science in Space Environment) Research project in the design and realization of two experiments to investigate in-tube condensation and condensation on fins onboard the ISS. Experiments concerning in-tube flow condensation and heat transfer enhancement on microstructured surfaces are planned to be performed onboard the Tiangong Chinese Space Station after 2022.

## Summary and outlook

To the authors’ knowledge, a total of fourteen works (ten experimental studies, four review papers) dealing with the experimental investigation of filmwise condensation in microgravity conditions have been published, while experiments of dropwise condensation in a reduced gravity environment are not yet reported in the literature.

In-tube condensation heat transfer under microgravity is penalized compared to normal gravity conditions, especially when the vapor shear stress decreases (i.e. decreasing mass flux and vapor quality, increasing channel diameter). For instance, considering condensation tests with HFE-7000 inside a 3.38 mm inner diameter channel, the heat transfer penalization under microgravity is negligible at mass velocity higher than or equal to 170 kg m^−2^ s^−1^ while it ranges from 50% to 80% at mass velocity equal to 30 kg m^−2^ s^−1^. Apart from the case of vapor shear driven condensation, the condensate can be removed in a microgravity environment by means of wall suction, surface tension, centrifugal or capillary forces and electromagnetic field.

The predictive tools for filmwise condensation heat transfer inside tubes generally consist of mechanistic criteria for identifying the flow conditions for gravity-independent condensation and modified 1-*g* correlations for the evaluation of the condensation heat transfer coefficient. For instance, the minimum mass velocity required to overcome body force effects on flow condensation heat transfer of FC-72 inside a 11.89 mm inner diameter tube is around 420 kg m^−2^ s^−1^.

Surface tension forces exert a non-negligible effect during condensation on finned surfaces. In particular, the highest condensation heat fluxes (in the order of 100 kW m^−2^) under reduced gravity are obtained at the corner of the fin tip, where the liquid film thickness along the fin is at its minimum. The fin shape represents one of the key parameters that need to be optimized during the design phase to change the distribution of the condensate film along the fin surface and, hence, improve the condensation heat transfer.

With regard to dropwise condensation, the dependence on gravity for liquid removal may limit its application in low gravity space systems. Techniques for droplets removal, both active (such as non-negligible vapor velocity) and passive ones (i.e. based on the use of condensing surfaces with wettability gradients or micrometric/nanometric surface structure) represent viable solutions for exploiting the benefits of dropwise condensation also in microgravitational environments.

Due to the difficulties and the costs of performing extensive space-based experiments, steady-state experimental data in microgravity conditions are still limited and may lack of repeatability and accuracy. Therefore, additional experimental and theoretical studies are required to better understand the condensation heat transfer mechanisms in space applications and for the development of reliable predicting tools for two-phase heat transport systems for thermal management in microgravity environments.

## Supplementary information

Supplementary information

## Data Availability

The data presented in this paper can be made available to the reader upon request to the corresponding author.
