# Supplementary material for: Condensation heat transfer in microgravity conditions
Source: NPJ Microgravity. 2023 Apr 4;9:32. doi: 10.1038/s41526-023-00276-1 (PMC10073138; doi:10.1038/s41526-023-00276-1)
Supplement: Supplementary file 1 — Supplementary information [file 41526_2023_276_MOESM1_ESM.docx]

**Supplementary information**

**A. Passive methods for condensate removal during filmwise condensation**

In a reduced gravity environment, the condensate liquid film must be drained away from the heat transfer surface to prevent condenser flooding and stoppage of the vapor flow^1^. Apart from vapor shear stress, the condensate can be removed in microgravity conditions by means of suction through a porous wall, capillary or centrifugal forces and electromagnetic fields. Limited experimental and numerical studies are available in the literature on this topic. Chow and Parish^2^ addressed two of these mechanisms for condensate removal in microgravity environments: the first one involving film condensation on a flat porous plate with the condensate being removed by suction at the wall, the second dealing with the analytical prediction of the heat transfer coefficient for condensing annular flows with the condensate film driven by the vapor shear. The authors concluded that both suction and vapor shear can effectively drain the condensate to ensure continuous operation of the condensers under microgravity. Faghri and Chow^3,4^ numerically studied forced condensation of vapor within a tube with uniform suction at the wall in a microgravitational environment. Increased heat transfer rates were obtained thanks to suction due to the reduction of the film thickness, especially for high values of the Reynolds number. Ma et al.^5^ developed a mathematical model to investigate the influence of the porous layer characteristics on the condensation heat transfer. The results revealed that the heat transfer enhancement due to wall suction is higher with the increasing thickness and permeability of the porous layer. Yang^6^ addressed the problem of film condensation on a porous wall by means of boundary layer theory and concluded that uniform suction causes a substantial increase of the heat transfer and condensation rate, particularly at low subcooling and at high Prandtl number. Renken and Aboye^7^ experimentally investigated the filmwise condensation process under reduced gravity occurring over a thin, permeable and highly conductive porous coating which was bonded to an isothermal copper block. The thin porous coating was found to produce a significant increase (up to 56%) in the heat transfer rate during condensation as compared to a plain surface due to the reduction in the liquid film thickness induced by suction. Adera et al.^8^ observed rapid condensate transport through the cracks formed on copper tubes coated with silica inverse opals (8-12 µm thickness), with nearly a sevenfold increase in the heat transfer performance compared to laminar filmwise condensation on smooth tubes.

Gravity can be also replaced during condensation by the artificial body forces due to the centrifugal forces generated by a rotating system^1^. Sparrow and Gregg^9^ performed preliminary studies of filmwise condensation of pure saturated vapor on a rotating disk and concluded that the centrifugal field associated to the rotation of the plate acts for sweeping the condensate outward the disk surface. The local condensation heat transfer *q’’* was found to be proportional to the square root of the disk angular velocity *ω*:

|  | $q''\propto\omega^{0.5}$ | (A1) |
| --- | --- | --- |

Mochizuki and Shiratori^10^ analytically investigated the effect of the centrifugal acceleration on the condensation heat transfer of steam within a vertical circular tube rotating around an axis parallel to its own. The results of the analytical study indicate that the overall heat transfer coefficient is substantially enhanced with the increasing centrifugal acceleration. Peng et al.^11^ experimentally studied filmwise condensation of R113 inside a horizontally rotating cylinder of 312 mm inner diameter with a scraper. The heat transfer coefficient was found to increase monotonically as the rotational speed was augmented in the presence of thin films, while it first increased and then decreased for thick films as the rotational speed became higher. These experimental results were confirmed by Alasadi et al.^12^, who conducted experimental condensation tests of steam on a rotating disk condenser at different rotational speed (103-150 rpm). Similarly, Al-Baroudi and Klein^13^ designed and tested a heat rejection system using condensation of superheated steam on a rotating flat plate and detected the optimal rotational speed for the desired heat rejection load.

Another way of reducing the effects of gravity relies on the decrease of the channel diameter. Owing to the miniaturization of condensers (diameters between 100 μm and 1 mm), the effect of the gravity force is reduced and the distribution of the phases is driven by the competition between capillary and viscous forces. Indeed, it is well documented that gravitational forces have a little significance at diameter size *D_h_* ≤ 1 mm^14,15^. Coleman and Garimella^16,17^ experimentally observed flow regimes during air-water adiabatic flow and with R134a condensing at 51.7 °C in horizontal tubes and square minichannels with hydraulic diameters ranging from 4.91 to 1 mm. As the hydraulic diameter decreases, the overall range of the annular and intermittent flow regime increases, while the wavy regime completely disappears at *D_h_* = 1 mm, showing a diminishing influence of the gravity force. From this point of view, the situation is equivalent to that found in microgravity. Miscevic et al.^18^ performed condensation experiments with n-pentane using a circular channel of 560 μm diameter. At low mass fluxes (lower than 7 kg m^-2^ s^-1^) the condensate takes the shape of an extended meniscus and heat transfer during capillary regime is very effective on the inner side of the heat exchanger. After reaching a critical flow rate (around 8 kg m^-2^ s^-1^), the meniscus breaks up producing a train of bubbles which lead to a 4-fold smaller condensation heat transfer coefficient compared to the case of low mass flux. The authors also developed a 1D model of convective condensation in the absence of gravity inside a capillary-driven two-phase loop at low mass flux. In particular, the numerical results suggest that the steady state no longer exists as the flow rate increases and intrinsic instabilities can occur in the condenser.

Wang and Antao^19^ proposed a model to study capillary-enhanced filmwise condensation in a high thermal conductivity porous wick to promote condensate removal. The model results indicate that increased wick thickness and permeability are linked to higher heat transfer coefficients and delay the failure that may occur when the condensate floods the wick. Chang et al.^20^ performed a theoretical investigation on laminar filmwise condensation over a horizontal semi-circular tube embedded in a porous medium and subject to capillary forces. The authors pointed out that, if the capillary force is greater than the condensate gravity force, the condensate can be sucked into the two-phase zone leading to a reduction of the liquid film thickness and to an improvement of the condensation heat transfer performance.

The condensation heat transfer can be further enhanced by means of electromagnetic fields, overcoming the limitations induced by the absence of gravity. Limited research has been carried out in the literature to investigate such effect on the condensation heat transfer, mainly on an experimental basis. Didkovsky and Bologa^21^ measured a twenty-fold increase in the heat transfer coefficient during condensation of stagnant pure vapor on a vertical surface exposed to an electric field of different strength, frequency and uniformity. As pointed out by Bologa et al.^22,23^, the enhancement of the condensation heat transfer under the electrohydrodynamic effect is due to the reduction of the film thickness caused by condensate spraying into the vapor phase and the formation of transverse waves.

**B. Experimental and numerical studies on heat pipes under reduced gravity**

Heat pipes have gained more and more relevance in the area of spacecraft cooling because of their lightweight, high performance and reliability^24^. Various heat pipes (Grooved Heat Pipes, Sintered Heat Pipes, Loop Heat Pipes, Capillary Pumped Loops) have been already qualified as “flight proven” through successful operations in Space missions^25^. Pulsating Heat Pipes, consisting of a small diameter tube bended in several turns with alternated heated and cooled zones, have been recently introduced and tested in reduced gravity environment due to the greater compactness and simpler design compared to standard heat pipes, as they do not require a wick structure. Experimental investigations during Parabolic Flights have been performed by Ayel et al.^26^ using a flat plate Pulsating Heat Pipe with square channel (2.5 x 2.5 mm^2^) filled with FC-72, by Mangini et al.^27^, Mameli et al.^28^ and Abela et al.^29^ using a 3 mm inner diameter tubular Pulsating Heat Pipe operating with FC-72. In all such experimental studies the device responds to the occurrence of the microgravity phase with a flow pattern transition from stratified to slug-plug regime and the flow pressure signal displays an oscillating trend throughout the entire 0-g period. Cecere et al.^30^ tested a “self-rewetting” water/butanol mixture (i.e. a fluid whose surface tension is an increasing function of the temperature that, in presence of a thermal gradient, provides a reverse Marangoni flow driven towards the hotter region of the liquid-vapor interface) in a single groove heat pipe, embedded in a semi-transparent test cell that allows the qualitative visualization of the liquid distribution along the channel during a Parabolic Flight. The potential of “self-rewetting fluids” was also assessed by Savino et al.^31^ from experimental tests carried out during a Parabolic Flight with different heat pipes, with composite wick or wickless, filled with pure water or with water/alcohol binary mixtures. The results showed that the heat pipes filled with binary mixtures are more efficient when compared to the same heat pipes filled with pure water owing to the delayed dry-out inception, and their performance is almost the same in normal gravity and low-gravity conditions.

Due to thermal inertia and working conditions, it was not always feasible to achieve a steady state condition during the aforementioned experimental studies, since the averaged value of the temperature and pressure still varied within the time lag of the microgravity period. Therefore, a long-term microgravity environment (i.e. suborbital flights, satellites, international space stations) is necessary to evaluate the actual performance of this kind of heat pipes at a pseudo-steady state.

Regarding numerical studies, in the work by Abela et al.^29^ a 1-D transient code was used to model the start-up of a large diameter Pulsating Heat Pipe and the results were compared against experimental data obtained during a Parabolic Flight campaign. The simulation results well agree with the experiment for different input powers, with temperatures predicted with a maximum deviation of 7% and an acceptable qualitative prediction of the pressure variation trend. Cecere et al.^30^ simulated the capillary flow of a water/butanol mixture in a single groove heat pipe under reduced gravity by means of CFD analysis. For a power input of 30 W, the authors detected a maximum deviation of 1.5 K between predicted and experimental temperatures in the test cell. Manzoni et al.^32^ developed a 1-D hybrid lumped parameter code for the transient simulation of pulsating heat pipes. The numerical code is able to reproduce with high accuracy both the stationary and the transient evolution of the local spatial average temperature that the pulsating heat pipe experiences during a parabolic flight.

**References**

1. Chen, Y., Sobhan, C. B. & Peterson, G. P. Review of condensation heat transfer in microgravity environments. *J. Thermophys. Heat Transf.* **20**, 353–360 (2006).

2. Chow, L. C. & Parish, R. C. Condensation heat transfer in a microgravity environment. *J. Thermophys. Tech. Notes* **2**, 82–84 (1988).

3. Faghri, A. & Chow, L. C. Forced condensation in a tube with suction at the wall for microgravitational applications. *J. Heat Transfer* **110**, 982–985 (1988).

4. Faghri, A. & Chow, L. C. Annular condensation heat transfer in a microgravity environment. *Int. Commun. Heat Mass Transf.* **18**, 715–729 (1991).

5. Ma, X. H., Chen, J. Bin & Wang, B. X. Interfacial suction effects of a porous layer on filmwise condensation heat transfer enhancement. *Heat Transf. - Asian Res.* **31**, 568–577 (2002).

6. Yang, J. W. Effect of Uniform Suction on Laminar Film Condensation on a Porous Vertical Wall. *Trans. ASME, Pap. No. 69-WA/HT-14* 252–256 (1970).

7. Renken, K. J. & Aboye, M. Experiments on film condensation promotion within thin inclined porous coatings. *Int. J. Heat Mass Transf.* **36**, 1347–1355 (1993).

8. Adera, S. *et al.* Enhanced condensation heat transfer using porous silica inverse opal coatings on copper tubes. *Sci. Rep.* **11**, 1–11 (2021).

9. Sparrow, E. M. & Gregg, J. L. The Effect of Vapor Drag on Rotating Condensation. *J. Heat Transfer* **82**, 71–72 (1960).

10. Mochizuki, S. & Shiratori, T. Condensation Heat Transfer Within a Circular Tube Under Centrifugal Acceleration Field. *J. Heat Transfer* **102**, 158–162 (1980).

11. Peng, S. W., Mizukami, K., Liu, W., Sebe, N. & Takeba, T. An experimental study of condensation heat transfer in a horizontally rotating cylinder with a scraper. *Exp. Therm. Fluid Sci.* **14**, 205–212 (1997).

12. Alasadi, A. A.-M., Ezzat, A. W. & Munner, A. Investigation of Steam Condensation Process on Rotating Disk Condenser at Different Rotation Speed. *Int. J. Comput. Appl.* **84**, 10–18 (2013).

13. Al-Baroudi, H. M. & Klein, A. C. Experimental simulations and heat transfer parameter measurements of film condensation on a rotating flat plate. *Exp. Therm. Fluid Sci.* **10**, 124–135 (1995).

14. Garimella, S., Killion, J. D. & Coleman, J. W. Experimentally validated model for two-phase pressure drop in the intermittent flow regime for circular microchannels. *J. Fluids Eng. Trans. ASME* **124**, 205–214 (2002).

15. Kandlikar, S. G., Garimella, S., Li, D., Colin, S. & King, M. R. *Heat transfer and fluid flow in minichannels and microchannels*. (Butterworth-Heinemann, 2013).

16. Coleman, J. W. & Garimella, S. Characterization of two-phase flow patterns in small diameter round and rectangular tubes. *Int. J. Heat Mass Transf.* **42**, 2869–2881 (1999).

17. Coleman, J. W. & Garimella, S. Two-Phase Flow Regime Transitions in Microchannel Tubes: The Effect of Hydraulic Diameter. In: *Proceedings of the ASME 2000 International Mechanical Engineering Congress and Exposition* vol. 4 71–83 (2000).

18. Miscevic, M., Médéric, B., Lavieille, P., Soupremanien, U. & Serin, V. Condensation in Capillary-Driven Two- Phase Loops. *Microgravity Sci. Technol.* **19**, 116–120 (2007).

19. Wang, R. & Antao, D. S. Capillary-Enhanced Filmwise Condensation in Porous Media. *Langmuir* **34**, 13855–13863 (2018).

20. Chang, T. B., Shiue, B. H., Ciou, Y. Bin & Lo, W. I. Analytical Investigation into Effects of Capillary Force on Condensate Film Flowing over Horizontal Semicircular Tube in Porous Medium. *Math. Probl. Eng.* **2021**, (2021).

21. Didkovsky, A. B. & Bologa, M. K. Vapour film condensation heat transfer and hydrodynamics under the influence of an electric field. *Int. J. Heat Mass Transf.* **24**, 811–819 (1981).

22. Bologa, M. K., Savin, I. K. & Didkovsky, A. B. Electric-field-induced enhancement of vapour condensation heat transfer in the presence of a non-condensable gas. *Int. J. Heat Mass Transf.* **30**, 1577–1585 (1987).

23. Bologa, M. K., Grosu, F. P., Polikarpov, A. A. & Motorin, O. V. Condensation of a gas-vapor mixture in an electric field. *Surf. Eng. Appl. Electrochem.* **45**, 125–127 (2009).

24. Butler, D., Ku, J. & Swanson, T. Loop heat pipes and capillary pumped loops-an applications perspective. In: *AIP Conference Proceedings 608* (2002). doi:10.1063/1.1449707.

25. Shukla, K. N. Heat Pipe for Aerospace Applications-An Overview. *J. Electron. Cool. Therm. Control* **5**, 1–14 (2015).

26. Ayel, V. *et al.* Visualization of Flow Patterns in Closed Loop Flat Plate Pulsating Heat Pipe Acting as Hybrid Thermosyphons under Various Gravity Levels. *Heat Transf. Eng.* **40**, 227–237 (2019).

27. Mangini, D. *et al.* A pulsating heat pipe for space applications: Ground and microgravity experiments. *Int. J. Therm. Sci.* **95**, 53–63 (2015).

28. Mameli, M. *et al.* Start-up in microgravity and local thermodynamic states of a hybrid loop thermosyphon/pulsating heat pipe. *Appl. Therm. Eng.* **158**, 113771 (2019).

29. Abela, M., Mameli, M., Nikolayev, V. & Filippeschi, S. Experimental analysis and transient numerical simulation of a large diameter pulsating heat pipe in microgravity conditions. *Int. J. Heat Mass Transf.* **187**, 122532 (2022).

30. Cecere, A., Di Martino, G. D. & Mungiguerra, S. Experimental Investigation of Capillary-Driven Two-Phase Flow in Water/Butanol under Reduced Gravity Conditions. *Microgravity Sci. Technol.* **31**, 425–434 (2019).

31. Savino, R., Abe, Y. & Fortezza, R. Comparative study of heat pipes with different working fluids under normal gravity and microgravity conditions. *Acta Astronaut.* **63**, 24–34 (2008).

32. Manzoni, M. *et al.* Advanced numerical method for a thermally induced slug flow: Application to a capillary closed loop pulsating heat pipe. *Int. J. Numer. Methods Fluids* **82**, 375–397 (2016).
